# Hæmatemesis Associated with Small White Kidneys

**Published:** 1904-09

**Authors:** Theodore Fisher

**Affiliations:** Pathologist to the Bristol Royal Infirmary; Physician to Out-Patients to the Bristol Royal Hospital for Sick Children and Women


### HÆMATEMESIS ASSOCIATED WITH SMALL WHITE KIDNEYS.

BY

THEODORE FISHER, M.D., M.R.C.P.,

*Pathologist to the Bristol Royal Infirmary; Physician to Out-Patients to the Bristol Royal Hospital for Sick Children and Women.*

INSTANCES of bleeding from the stomach unassociated with gross lesions of the gastric mucous membrane are always interesting, and often difficult of explanation. In the following cases 'small white kidneys' were found after death where there had been a history of hæmatemesis during life, and although the connection between this form of nephritis and hæmorrhage

1 *Lancet*, 1900, ii. 861, 932. 2 *Tr. Path. Soc. Lond.*, 1901, lii. 248.
3 *J. Path. and Bacteriol.*, 1904, ix. 276.

from the stomach may not be clear, the association is at least worthy of note.

**Case 1.**—Matilda F., aged 37, was admitted to the Bristol Royal Infirmary under the care of Dr. Prowse, October 29th, 1895, with a history of having, five weeks before, vomited blood, and on several occasions after that time. There was occasional diarrhœa. Five years before she had had scarlet fever, but there was no history of dropsy following. She died four days later.

On admission the patient was very drowsy, and an erythematous, patchy rash was present over the greater part of the body. The sp. gr. of the urine was 1015; .02 per cent. of albumin was present.

In the *post-mortem* room the fact that no œdema of the legs was present was noted. A careful search was made for the source of the hæmatemesis, but none could be found in either the stomach or the œsophagus. The liver was healthy; the heart weighed 7 oz., and presented nothing abnormal. The kidneys were both very small, the right weighed $2\frac{3}{4}$ oz. and the left $2\frac{1}{2}$ oz.; they were granular, pale, and had the speckled appearance due to opaque yellowish-white spots dotting a slightly darker and more transparent surface which is frequently seen in small white kidneys; the capsule also, as is not uncommonly the case, peeled without tearing the kidney substance; the cortex was much diminished, and in places had almost disappeared.

**Case 2.**—Caroline H., aged 45, was admitted into the Bristol Royal Infirmary, under the care of Dr. Prowse, July 7th, 1897. She was said to have enjoyed good health until four years before, when she had her first attack of bad dyspepsia. Shortly before admission it had returned, and on the day of admission she vomited about "half a gallon of blood."

On admission she was described as anæmic. The urine was said to contain no albumin; the sp. gr. was 1015. On July 13th, six days later, blood was passed per rectum, in quantity described as a "considerable hæmorrhage." She died July 27th. There was slight pyrexia during the time she was in the Infirmary.

At the necropsy "careful examination of the stomach failed to reveal anything that could account for the hæmatemesis. There was a doubtful scar of an old ulcer, but the mucous membrane of the stomach seemed remarkably healthy. The œsophagus showed nothing that could account for the hæmorrhage; the veins were unusually small. The duodenum was also healthy, as was the whole length of the small and large intestine." The liver showed no trace of cirrhosis, but was pale and apparently slightly fatty. The kidneys were of the 'small white' variety; the right weighed $2\frac{1}{2}$ oz., the left 2 oz.; the cortex of each was much diminished, and the capsule peeled badly.

As in the preceding case, the heart was not enlarged it weighed 7 oz.

**Case 3.**—Amelia D., aged 46, was admitted into the Bristol Royal Infirmary, under the care of Dr. Shingleton Smith, October 21st, 1897. She had suffered from dyspeptic symptoms for six months, and ten days before admission she vomited blood, and again nine days before. The motions at the time of the vomiting had been black.

On admission the patient was very anæmic, and was drowsy and apathetic. The notes make no mention of œdema of the legs. She died four days later. Slight pyrexia had been present.

At the autopsy nothing could be found in the stomach or œsophagus to account for the hæmatemesis. The liver was healthy. The kidneys were of the 'small white' variety; the weights were $2\frac{3}{4}$ and $2\frac{1}{2}$ oz. The heart weighed $10\frac{1}{2}$ oz.

**Case 4.**—W. M. C., aged 52, was admitted into the Bristol Royal Infirmary, under the care of Dr. Shaw, June 19th, 1896. He had suffered from repeated attacks of hæmatemesis shortly before admission.

At the time of admission he was very drowsy, and on the first day once vomited "coffee-ground" vomit. Two days later he died.

In the notes of the necropsy the stomach is described as "perfectly healthy, not even congested. There were no varicose veins of the œsophagus from which the blood could have come. There was no duodenal ulcer, and the intestines were healthy throughout." The liver showed no signs of cirrhosis, and the kidneys were described as small, both "very distinctly granular, but pale; the capsule peeled without tearing the kidney substance, and the peripheral cortex was much diminished." The heart weighed $8\frac{1}{2}$ oz.

The age of this patient may suggest that the kidneys were of the red granular type. Although, however, in the description of them the words 'small white kidney' are not used, it is obvious that they were considered to be examples of that form of diseased kidney, because the notes state: "As is usual with this variety of granular kidney, there was no arterio-sclerosis worth mentioning and no enlargement of the heart."

The following case is of somewhat different character. There was hæmoptysis but not hæmatemesis. The fact, however, that there was loss of blood for which no cause could be found associated with the presence of 'small white' kidneys justifies brief notes being inserted here.

**Case 5.**—David T., aged 20, was admitted under the care of Dr. J. E. Shaw, May 12th, 1890. He was said never to have had "a day's illness in his life" until three weeks before admission, up till which time he had assisted his father in a blacksmith's forge. He had felt languid for three weeks, and had suffered from a "bilious attack" ten days before. On the day of admission he coughed up "a quantity of blood," and during the first week he was in the Bristol Royal Infirmary there were several slight recurrences of the hæmoptysis.

At the time of admission he was a muscular young man. His urine was found to contain albumin, but there was no œdema of the legs. The urine was tested daily during his stay in the Infirmary. The amount of albumin was generally small, not more than .025 per cent., but occasionally rose to .15. The sp. gr. was generally about 1010. He grew gradually worse, became very emaciated, and for a month was covered with a patchy erythematous rash which desquamated. He died three months after admission.

At the necropsy nothing was found that could account for the hæmoptysis. The lungs were merely anæmic and the pleuræ were quite healthy, there being no evidence of even very slight tuberculous disease. The kidneys were both well-marked examples of the 'small white kidney,' but not so small as some of those in the preceding cases. Each weighed $3\frac{3}{4}$ oz. The peripheral cortex, however, had almost disappeared. The heart weighed $14\frac{1}{2}$ oz.

I have met with at least three other cases where severe bleeding from the stomach occurred shortly before death, yet I was unable to find a cause for the hæmorrhage. In one case an exploratory operation had been performed on the stomach on account of repeated hæmorrhages. She, however, died from a recurrence of the bleeding, yet no point of origin for the loss of blood could be found in the stomach or œsophagus, nor was any abnormal condition of the portal vein or liver present which would have accounted for it.

I am aware that very minute erosions of the gastric mucous membrane have been recorded in cases of fatal hæmorrhage, but feel sure that nothing of the kind was overlooked. And we know that hæmorrhages occur from the mucous surfaces in various forms of purpura where there is no reason to suppose that local lesions of the mucous membrane are present. In diphtheria also, where there had been hæmatemesis during life, I have been unable to find anything but minute circular hæmorrhages in the mucous membrane, without, however, any abrasion of the surface such as is seen in hæmorrhagic erosions.

It has been suggested that some toxins, like a few drugs such as morphia, may be excreted by the stomach. The appearance of the stomach in the case of diphtheria was certainly suggestive of the action of some substance which affected its mucous membrane more than any other surface of the body, but, curiously enough, not the stomach only. The whole of the small intestine was free from a single spot, but petechiæ similar to those seen in the stomach were scattered through the mucous membrane of the large intestine.

Whether, however, some toxins may or may not have some deleterious influence especially upon the stomach, it is interesting to note that some poisons appear to have an affinity for the endothelium of blood-vessels, and by weakening the wall allow of extravasation of blood. Such poisons, for which Flexner has proposed the name of "hæmorrhagins,"[1] may possibly sometimes exist in cases of Bright's disease, and be the explanation of hæmorrhages such as hæmatemesis.

In conclusion, it may be remarked that although hæmatemesis is mentioned amongst the complications of Bright's disease, I have not been able to find that it has been mentioned to occur especially in cases of the 'small white' kidney. It has so happened, however, that the only form of diseased kidney which I have met with in association with hæmatemesis has been of this variety.

